# FPGA-Based High-Performance Phonocardiography System for Extraction of Cardiac Sound Components Using Inverse Delayed Neuron Model

**DOI:** 10.3389/fmedt.2021.666650

**Published:** 2021-08-12

**Authors:** Madhubabu Anumukonda, Prasadraju Lakkamraju, Shubhajit Roy Chowdhury

**Affiliations:** ^1^Center for Very Large Scale Integration and Embedded Systems Technology, International Institute of Information Technology Hyderabad, Hyderabad, India; ^2^School for Computing and Electrical Engineering, Indian Institute of Technology Mandi, Suran, India

**Keywords:** phonocardiography, cardiac sounds, inverse delayed function model of neuron, artificial neural networks, field programmable gate array

## Abstract

The study focuses on the extraction of cardiac sound components using a multi-channel micro-electromechanical system (MEMS) microphone-based phonocardiography system. The proposed multi-channel phonocardiography system classifies the cardiac sound components using artificial neural networks (ANNs) and synaptic weights that are calculated using the inverse delayed (ID) function model of the neuron. The proposed ANN model was simulated in MATLAB^R^ and implemented in a field-programmable gate array (FPGA). The proposed system examined both abnormal and normal samples collected from 30 patients. Experimental results revealed a good sensitivity of 99.1% and an accuracy of 0.9.

## Introduction

Heart diseases are one of the major causes of human death worldwide. In the last 15 years, heart disease and stroke have been the leading killers and causes of death on a global scale (1). Heart failure has no cure, but early detection of its related symptoms helps in properly diagnosing heart diseases and, thus, reducing the death rate. In the modern technological revolution, many heart diagnosis methods like phonocardiography (PCG), ECG, echocardiogram (Echo), cardiac MRI (CMRI), and CT heart scan are available to detect early heart failure. Each method has its own advantages and disadvantages; for example, ECGs are widely used in diagnosis but have trouble detecting the structural abnormalities of the heart valves, which can be detected through heart murmurs. Furthermore, echo, CMRI, and CT scans provide accurate results but need a lot of pre-evaluation, are high cost, and are not affordable for many people.

Phonocardiography is one of the non-invasive methods for the detection of major heart sounds and murmurs (2). The stethoscope was the primary PCG instrument that was used to detect cardiac auscultation; however, it had limitations in terms of clinical expertise for the analysis of the low-frequency amplitudes formed during heart failures, such as systolic and diastolic murmurs (3, 4). A great deal of research has been conducted in the field of heart sound segmentation and classification in order to detect normal and abnormal components. Many signal processing algorithms are proposed to extract heart sound components for detecting pathological events (5–7). Artificial neural networks (ANNs) are widely used in cardiology (8–10). A neural network model is a well-known method for separating the normal and abnormal pathological events in heart sounds (11–14). A well-trained neural network model can be used to detect the complex relationship between abnormal and normal cardiac sounds. Deep neural networks extract high-level features from low-level features. The methods proposed (15–17) to play significant roles in solving the non-linear functions in medical applications.

Cardiac auscultations are non-linear in nature and are analyzed using ANNs, which are more useful for the approximation of non-linear functions. The neural networks models are mainly classified by their architecture, activation function, and learning algorithm. Instead of directly emulating biological behavior, the traditional neuron network models translate this behavior in terms of time-averaging techniques (9, 18, 19). The inverse delayed (ID) function model proposed by Nakajima (20–22) is a universal neuron model that includes characteristics of both the Bonhoeffer Van der Pol model (19) and the Hopfield model (23). In addition, the ID function model uses the inverse function of the activation function rather than the traditional activation function and features a finite conversion time from the internal state of the element to the output.

The energy function of the ID function model with symmetric synapse weights is similar to that of the Hopfield model. Through selective destabilization, the negative resistance of the ID model can free the neural network state from such local minima (20). Unlike the chaotic neural network, the ID model does not need to transform the output vector, record the output vector during calculation, or control the dynamics by changing the network parameters (22). We only need to wait for it to enter an inactive state in order to find a solution using the ID neural network and a simple method of implementation. The ID model is capable of resolving combinatorial optimization problems (21). The negative resistance of the ID model can destabilize the stable equilibrium points of a neural network, reducing the possibility of unknown values in suboptimal synaptic weight solutions obtained using an ANN based on a traditional neuron model. The ANN implementation using the ID neuron model needs numerous parallel computations to solve the real-time complex data for the extraction of components. The advances in field-programmable gate arrays (FPGAs) handle these real-time complex computations effectively and improve the performance of the system.

The current study concentrated on the non-invasive detection of cardiac component abnormalities in raw samples collected with micro-electromechanical system (MEMS)-based microphones. Wavelet decomposition algorithms were used to generate the featured set. The ID neuron function was used to create the ANN model, which extracted the cardiac components from the feature set obtained. The ID function model of the neuron was used to optimize the weights of the synapses between the neurons. The entire algorithm was implemented on a Xilinx SoC FPGA XC7Z020CLG400 (Xilinx, USA). The proposed system has been validated using the sensitivity, specificity, and accuracy of the cardiac components, and justified using receiver operating characteristic curve analysis. The study is organized as follows: Section Theory focuses on the theory supporting the proposed method using ID function model of the neuron and illustrates the feature sets for cardiac sound assessment. Section Materials and Methods focuses on the materials and methods for the proposed technology. The results and discussions are presented in Section Experimental Results and Discussion.

## Theory

### Inverse Delayed Neuron Function Model of the Neuron

The ANN was realized using the ID function model of the neuron. Nakajima and Hayakawa proposed the ID function model of the neuron by the following set of equations:


(1) $$\begin{array}{l} \tau \frac{\text{d}{\text{u}}_{\text{i}}}{\text{d}\text{t}}\;=\;\underset{\text{j}.\text{j}\neq \text{i}}{\sum }{{\text{w}}_{\text{i}\text{j}}\text{x}}_{\text{j}}-{\text{a}}_{\text{i}\text{i}}{\text{x}}_{\text{i}}-{\text{u}}_{\text{i}} \end{array}$$

(2) $$\begin{array}{l} \tau_{\text{x}}\frac{\text{d}{\text{x}}_{\text{i}}}{\text{d}\text{t}}\;=\;{\text{u}}_{\text{i}}-\text{g}\left({\text{x}}_{\text{i}}\right) \end{array}$$

(3) $$\begin{array}{l} \text{g}\left({\text{x}}_{\text{i}}\right)={\text{f}}^{-1}\left({\text{x}}_{\text{i}}\right)-\text{K}{\text{x}}_{\text{i}} \end{array}$$

Where u_i_ is the ith neuron internal state, x_j_ is the jth neuron output, W_ij_ is the synaptic weight between jth and ith neurons, h_*i*_ is the bias input, a_ii_ is the self-connection synaptic weight, τ is the internal state time constant, and τ_x_ is the neuron output time constant.

From Equation (3), f(x) is the neural network sigmoid function, then g(x) = f^−1^(x) is the N-shaped inverse output function (24). g(x) can be changed with a positive value of K times output of the neuron. The transition time from u to x is less than τ and thus τ_x_ < < τ. In general, the transition time should be taken into account if it is significantly less than τ. For the present problem, we used the self-connectionless neurons to devoid the hysteresis effects (24). So a_ii_ = 0 in Equation (1).

Differentiating Equation (2) with respect to time t, we get


(4) $$\begin{array}{l} \tau_{\text{x}}\frac{{\text{d}}^{2}{\text{x}}_{\text{i}}}{\text{d}{\text{t}}^{2}}=\frac{\text{d}{\text{u}}_{\text{i}}}{\text{d}\text{t}}-\frac{\text{d}\text{g}\left({\text{x}}_{\text{i}}\right)}{\text{d}{\text{x}}_{\text{i}}}\frac{\text{d}{\text{x}}_{\text{i}}}{\text{d}\text{t}} \end{array}$$

Let us consider


(5) $$\begin{array}{l} \varphi_{\text{i}}=\frac{\text{d}\text{g}\left({\text{x}}_{\text{i}}\right)}{\text{d}{\text{x}}_{\text{i}}}+\frac{\tau_{\text{x}}}{\tau }\; \end{array}$$

Substituting Equation (5) and (4) gives


(6) $$\begin{array}{l} \tau_{\text{x}}\frac{{\text{d}}^{2}{\text{x}}_{\text{i}}}{\text{d}{\text{t}}^{2}}+\varphi_{\text{i}}\frac{\text{d}{\text{x}}_{\text{i}}}{\text{dt}}-\frac{\tau_{\text{x}}}{\tau }\frac{\text{d}{\text{x}}_{\text{i}}}{\text{dt}}=\frac{\text{d}{\text{u}}_{\text{i}}}{\text{dt}}\Leftrightarrow \tau_{\text{x}}\frac{{\text{d}}^{2}{\text{x}}_{\text{i}}}{\text{d}{\text{t}}^{2}}+\varphi_{\text{i}}\frac{\text{d}{\text{x}}_{\text{i}}}{\text{dt}}\\ =\frac{1}{\tau }\left(\sum_{\text{j}}({\text{w}}_{\text{ij}}{\text{x}}_{\text{j}}-\text{g}\left({\text{x}}_{\text{i}}\right)\right) \end{array}$$

Let, $\frac{\partial U_{i}}{\partial x_{i}}=\frac{1}{\tau }\left(g\left(x_{i}\right)-\sum w_{ij}x_{j}\;\right)$

Equation (6) becomes


(7) $$\begin{array}{l} \tau_{\text{x}}\frac{{\text{d}}^{2}{\text{x}}_{\text{i}}}{\text{d}{\text{t}}^{2}}+\varphi_{\text{i}}\frac{\text{d}{\text{x}}_{\text{i}}}{\text{d}\text{t}}=\frac{\partial {\text{U}}_{\text{i}}}{\partial {\text{x}}_{\text{i}}} \end{array}$$

Where $U_{i}=\frac{1}{\tau }\left(\int_{0}^{x}g(x_{i}\right)dx_{i}-\;x_{i}\sum_{j}w_{ij}x_{j}$

*U*_*i*_ denotes the potential of the ID function model of the neuron. In Equation (7), the first term denotes the inertia and the second term denotes the friction. If g(x_i_) is an N-shaped function, then the area where

$\frac{dg\left(x\right)}{dx_{i}}$ is less than $\frac{-\tau_{x}}{\tau }$ for specific values of x_i_ is called the negative resistance region.

From the Lyapunov function, the energy of the ID function model is


(8) $$\begin{array}{l} \text{E}=-\frac{1}{2\tau }\sum_{\text{i}}\sum_{\text{j}}{\text{w}}_{\text{ij}}{\text{x}}_{\text{i}}{\text{x}}_{\text{j}}+\frac{1}{\tau }\sum_{\text{i}}\int_{0}\text{g}\left({\text{x}}_{\text{i}}\right)\text{d}{\text{x}}_{\text{i}}\\ +\frac{\tau_{\text{x}}}{2}\sum_{\text{i}}{\left(\frac{\text{d}{\text{x}}_{\text{i}}}{\text{dt}}\right)}^{2} \end{array}$$

Since the proposed neuron network has self-connectionless neurons, the self-connections between neurons are ignored. The last term in Equation (8) shows the time delay in the ID function model.

Differentiating both sides of Equation (8) with respect to time t, we get


(9) $$\begin{array}{l} \frac{\text{d}\text{E}}{\text{d}\text{t}}=-\underset{\text{i}}{\sum }\frac{\text{d}{\text{x}}_{\text{i}}}{\text{d}\text{t}}\;\left\{\frac{1}{\tau }{\text{w}}_{\text{i}\text{j}}{\text{x}}_{\text{j}}-\frac{1}{\tau }\text{g}\left({\text{x}}_{\text{i}}\right)-\tau_{\text{x}}\frac{{\text{d}}^{2}{\text{x}}_{\text{i}}}{\text{d}{\text{t}}^{2}}\right\} \end{array}$$

(10) $$\begin{array}{l} \frac{\text{d}\text{E}}{\text{d}\text{t}}=-\underset{\text{i}}{\sum }\left(\frac{\text{d}\text{g}\left({\text{x}}_{\text{i}}\right)}{\text{d}{\text{x}}_{\text{i}}}+\frac{\tau_{\text{x}}}{\tau }\right)\;{\left(\frac{\text{d}{\text{x}}_{\text{i}}}{\text{d}\text{t}}\right)}^{2} \end{array}$$

(11) $$\begin{array}{l} \frac{\text{dE}}{\text{dt}}=-\underset{\text{i}}{\sum }\varphi_{\text{i}}\;{\left(\frac{\text{d}{\text{x}}_{\text{i}}}{\text{dt}}\right)}^{2} \end{array}$$

From Equation (11), the energy (E) of the ID model, similar to that of the Hopfield model, decreases with time if the network state is in the positive resistance region (ϕi > 0). However, in the negative resistance region (ϕi < 0), the energy (E) increases with time; thus, even if the state is in the minima region, it quickly exits this region. It is necessary to have this feature in order to avoid local minima. As a result, if the network is an ID function model, the likelihood of escaping the local minima is expected to increase.

### Feature Sets for Cardiac Sound Assessment

The assessment of the cardiac sound components involved different parameters that were deferred by a set of features to sort out the components from the heart sounds. In the current research, the Springer segmentation algorithm by Springer et al. (25) was used to differentiate the heart sounds using timing intervals of S1, S2, systole, and diastole. The following parameters were used as feature sets to identify the low-frequency abnormal components and normal heart sound components.

## Materials and Methods

### High-Performance Phonocardiography System Hardware

**Figure 1:**
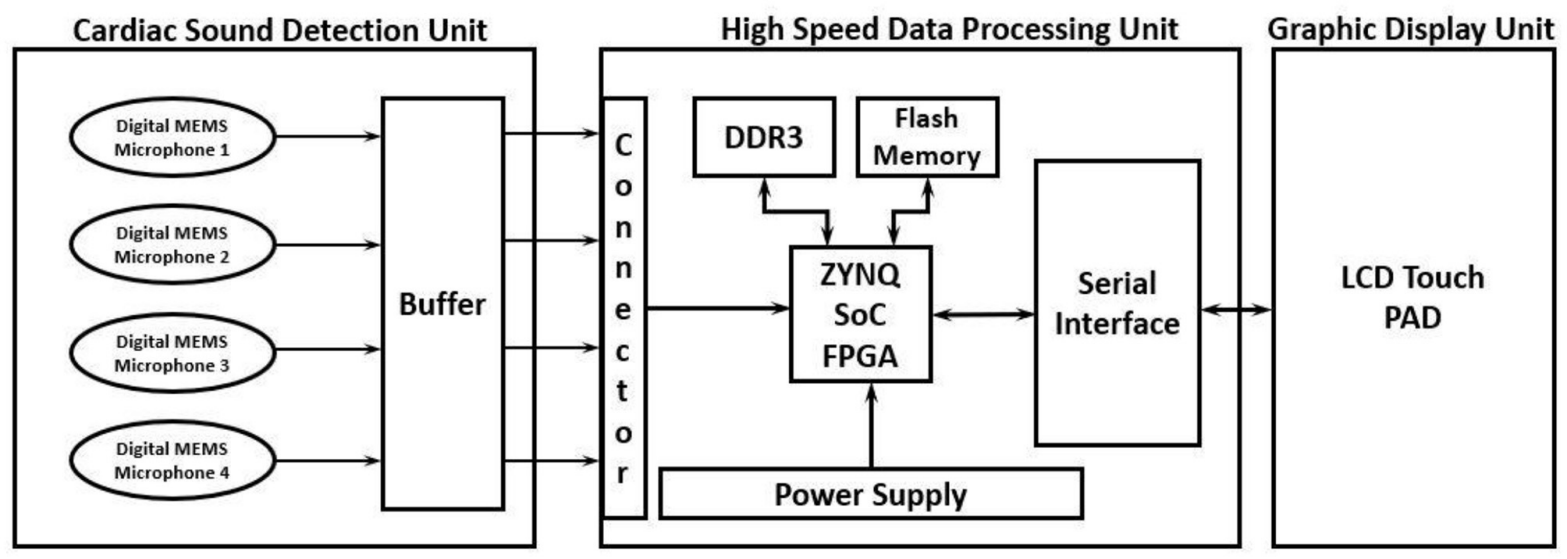
Phonocardiography system block diagram.

The proposed high-performance phonocardiography system was developed based on the advanced MEMS microphone to capture the low-frequency components and analyze the captured data using a proposed algorithm based on the inverse delayed neuron model. The proposed algorithm was implemented on the Xilinx Zynq-7 System on-chip FPGA, which has a dual-core ARM cortex-A9 for application software and programmable logic for algorithm complex computations. The detailed block diagram for the high-performance phonocardiography system is as shown in Figure 1.

**Figure 2:**
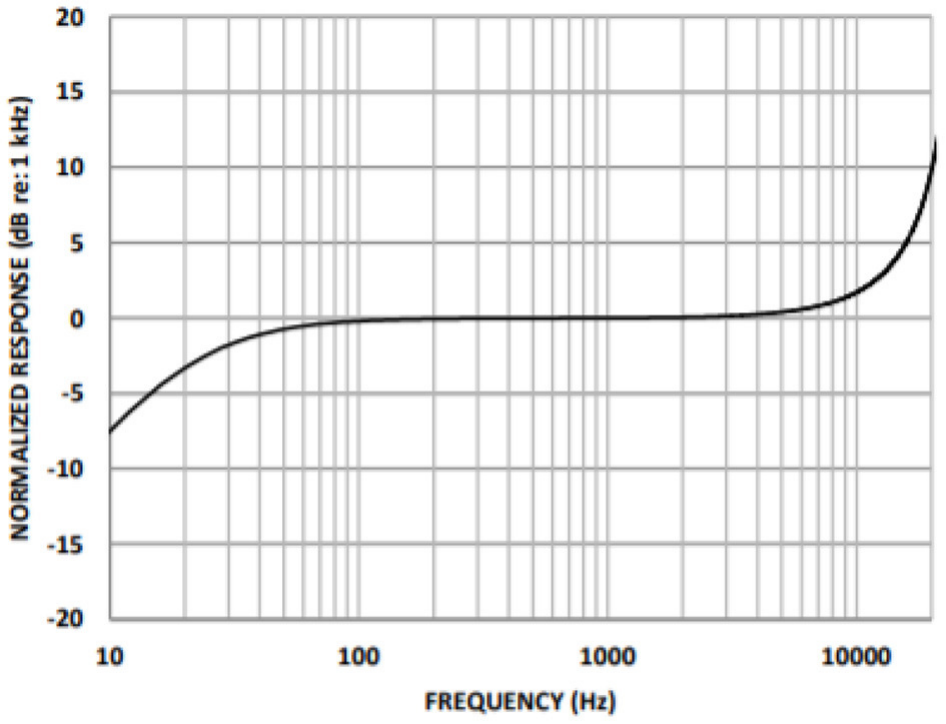
Micro-electromechanical system (MEMS) microphone frequency response.

A cardiac sound detection unit consists of a MEMS microphone, which is a tiny integrated circuit with a sound transducer, an analog front end, and a signal conditioning circuit (7). The MEMS microphone has a high Signal-to-Noise Ratio (SNR) at 70 dB and a good frequency response from 10 Hz to 10 kHz as shown in Figure 2. Due to its flat response and high SNR in the lower region, a cardiac sound detection (CSD) is more suitable for the detection of the third and fourth heart sounds and murmurs. The CSD module has four microphones, the placements of which were on the basis of sound source localization to cover the four heart valves (aortic valve, tricuspid valve, mitral valve, and pulmonary valve). These valves are the origins of cardiac sounds.

A high-speed data processing unit consists of a Xilinx Zynq-7 System on-chip FPGA, which has a dual-core ARM cortex-A9 processor for the application software and programmable logic for algorithm complex computations. The high-speed data processing (HSDP) module is responsible for the separation of cardiac sounds based on the frequencies and processes used in the proposed ANN-based ID model neuron. The Graphical Display Recorder (GDR) unit consists of an LCD touchpad for parameter configuration and a display for analyzing the results for further diagnosis. The application software was developed on an ARM cortex-A9 processor and interfaced to the GDR module.

The current system implemented was in two phases. In the first phase, the proposed algorithm was modeled using the MATLAB, simulated with different test parameters, and baselined as a golden reference for further hardware system development. The proposed neural network model was implemented on FPGA in the second phase to achieve performance comparable to the MATLAB model. The cardiac sounds detected by the CSD unit were then passed to the HSDP unit for the extraction of the cardiac feature set mentioned in Table 1. Afterward, these feature sets were given as inputs to the hidden layer and known spectrograms, which would train the network and predict the error deviation to assess the features of the cardiac sounds.

**Table 1:** Cardiac sounds feature sets.

S. no	Feature	Description
1	F1	Mean of the Systolic to diastolic time interval ratio of each heart sound.
2	F2	Mean of the S1, S2 intervals ratio.
3	F3	Mean of the heart sound peak energy in systolic cycle to total cardiac cycle energy of each heart beat
4	F4	Mean of the heart sound peak energy in diastolic cycle to total cardiac cycle energy of each heartbeat.
5	F5	Mean of spectral frequencies from 10 to 900 Hz with window resolution of 10 Hz in systole cycle of each heartbeat.
6	F6	Mean of spectral frequencies from 10 to 900 Hz with window resolution of 10 Hz in diastolic cycle of each heartbeat.

### Prediction Model Using ID Function Model of the Neuron

**Figure 3:**
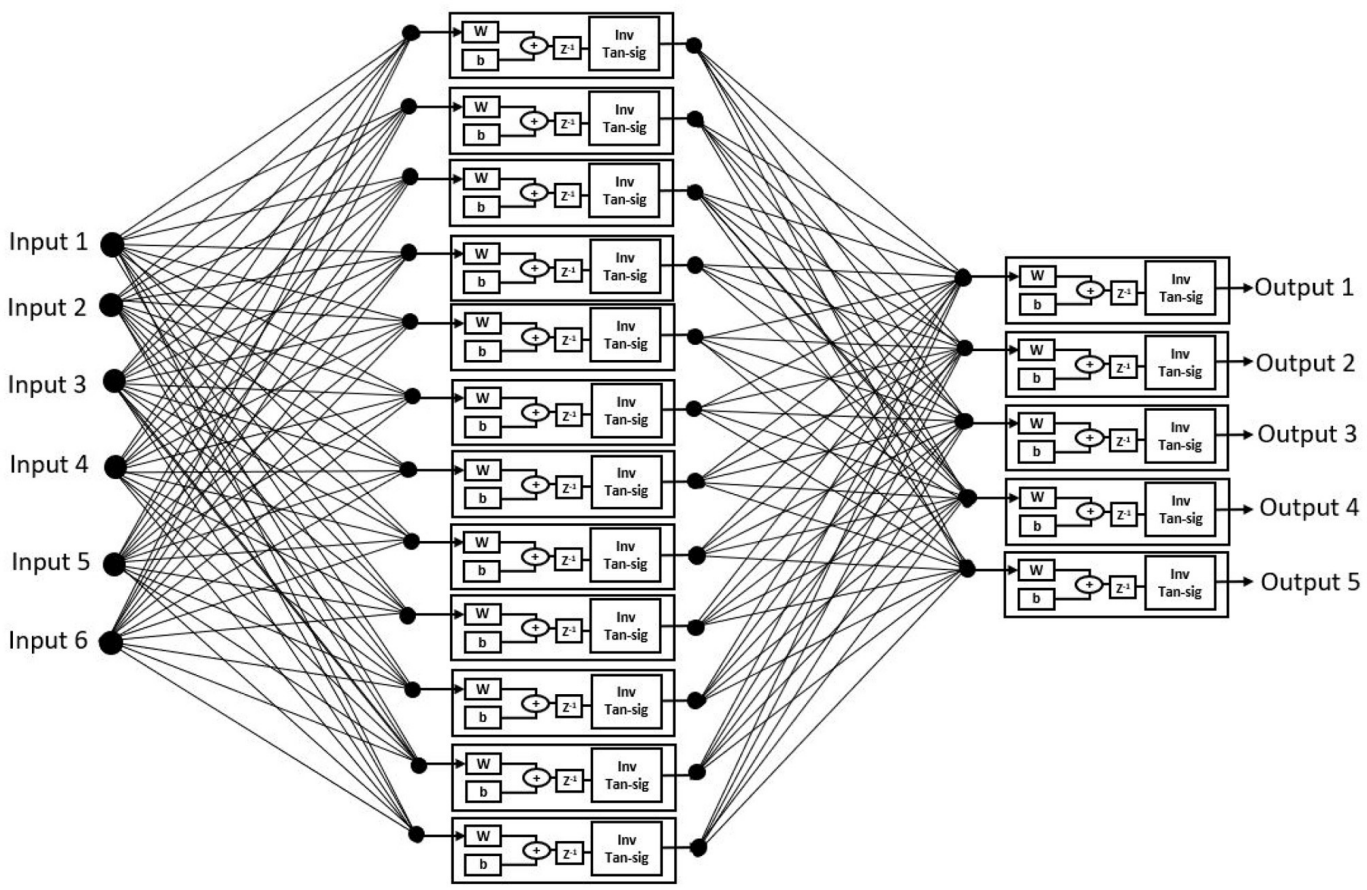
Artificial neural networks (ANNs) using the ID function neuron model.

The proposed ANN algorithm is based on a feedforward network with three layers as shown in Figure 3. The first layer has six inputs for the six feature sets mentioned in Table 1. The second layer is a hidden layer that consists of 12 hidden neurons that compute the delayed weighted sum of inputs and the inverse tangent sigmoid non-linear function for the feature extraction. The third layer is an output layer that consists of five output neurons. It is a logical net to reduce the error in extraction and sends the output based on the input from the hidden layer.

### Realization of Inverse Activation Function

An activation function is used to present non-linearity into the output of the neuron. For the current work, an inverse tan hyperbolic function is taken as the activation function.


(12) $$\begin{array}{l} \text{f}\left(\text{x}\right)=\text{ta}{\text{n}}^{-1}\text{h}\left(\text{x}\right)=\frac{1}{2}\text{ln}\left(\frac{1+\text{x}}{1-\text{x}}\right) \end{array}$$

(13) $$\begin{array}{l} \text{f}\left(\text{x}\right)=\frac{1}{2}(\text{ln}\left(1+\text{x}\right)-\text{ln}\left(1-\text{x}\right)) \end{array}$$

(14) $$\begin{array}{l} \text{f}\left(\text{x}\right)=\frac{1}{2}\left(2\text{x}+\frac{2}{3}{\text{x}}^{3}+\frac{2}{5}{\text{x}}^{5}+\ldots \right) \end{array}$$

(15) $$\begin{array}{l} \text{f}\left(\text{x}\right)=\text{x}+\frac{{\text{x}}^{3}}{3}+\frac{{\text{x}}^{5}}{5}+\ldots \end{array}$$

Neglecting the higher terms f(x) becomes


(16) $$\begin{array}{l} \text{f}\left(\text{x}\right)=x+\frac{{\text{x}}^{3}}{3} \end{array}$$

Equation (16) is realized using a constant (1/3), adder, and multiplier. The activation limits the output in the range of (1,−1).

**Figure 4:**
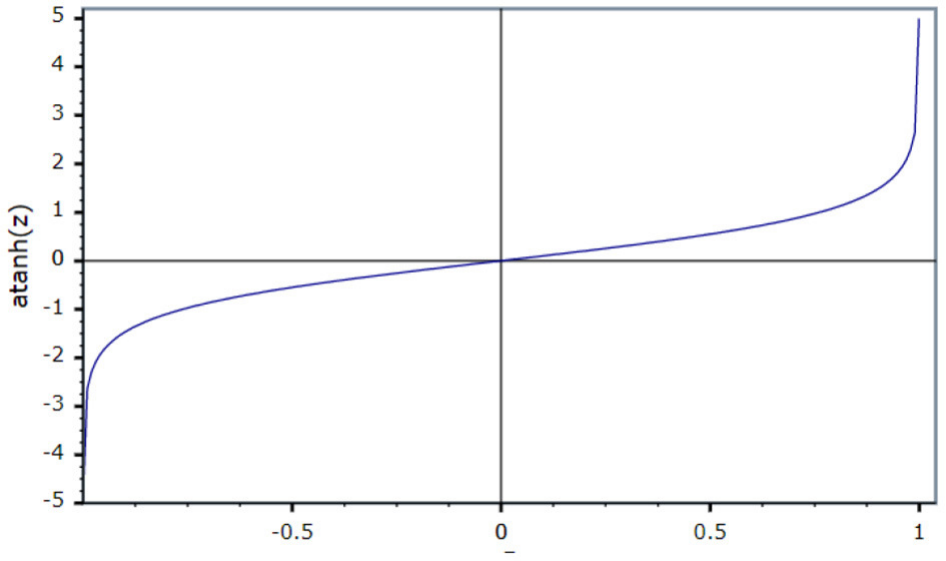
Transfer function.

Figure 4 shows that the activation function in the ID model is an N-shaped transfer function.

The ID network, which consisted of 12 hidden neurons in the hidden layer, was trained using MATLAB^R^. Learning was accomplished through the use of the Levenberg–Marquardt backpropagation algorithm. Backpropagation was used to obtain the input and layer weight matrices by incorporating the derivatives of the inverse functions. These matrices were used to replicate the ID network onto the FPGA. The neural network was trained using the ID function model. The result of the regression coefficient “*R*” is shown in **Figure 13**.

### System-Level Implementation

**Figure 5:**
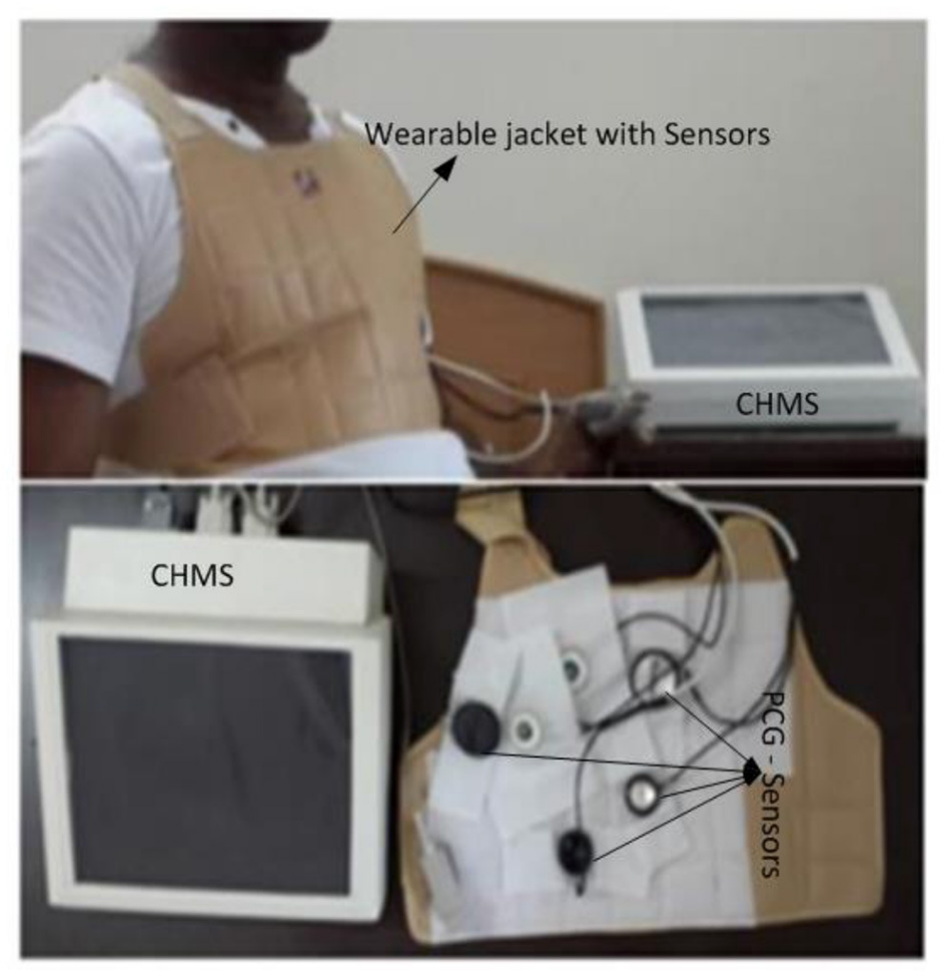
Real-time experimental setup with proposed hardware.

**Figure 6:**
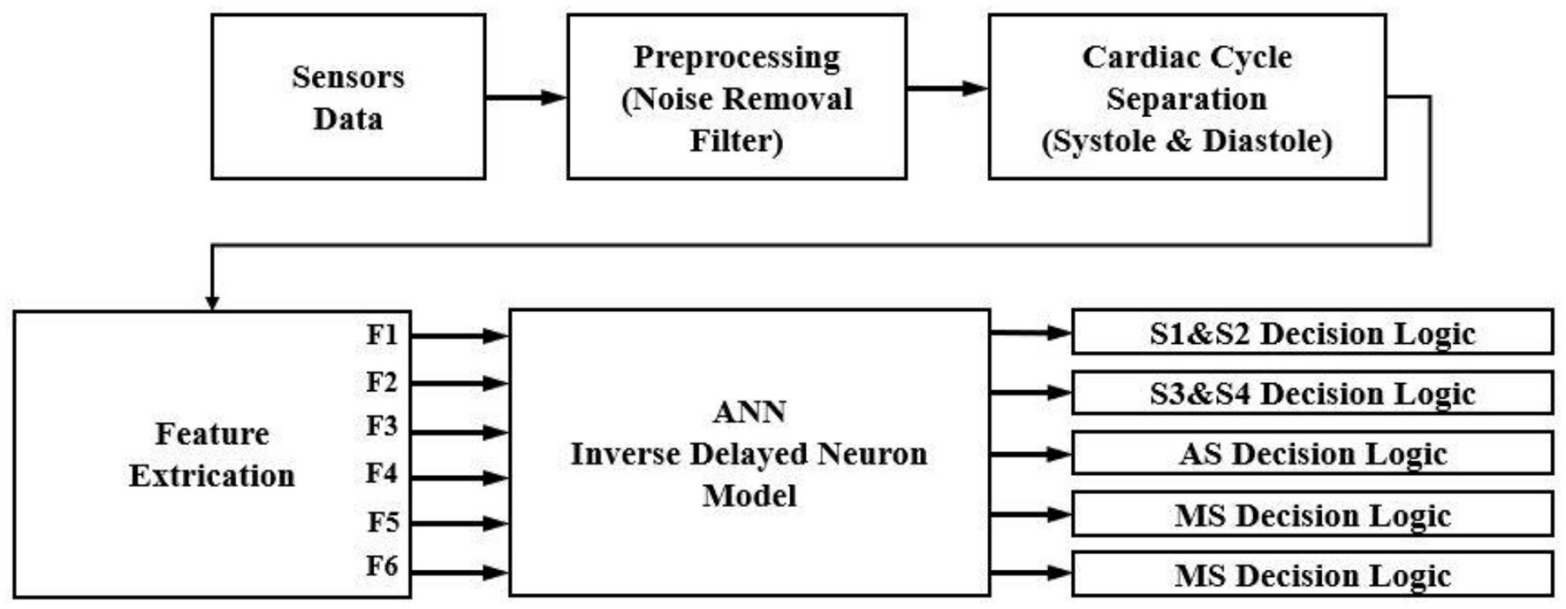
Flow diagram for extraction of cardiac sound components.

**Figure 7:**
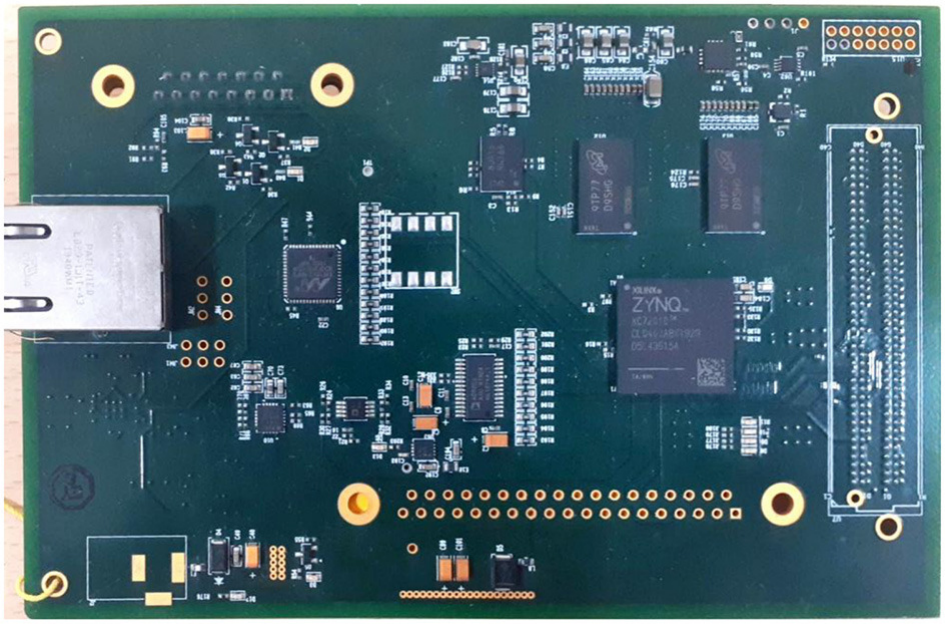
Field-programmable gate array (FPGA) hardware for the proposed system.

The experimental setup with PCG sensors and the Cardiac Health Monitoring System (CHMS) is shown in Figure 5. The proposed algorithm implementation in FPGA is shown in Figure 6. The prediction model, which was based on the ID function neuron model, was developed in the system generator tool, and the Verilog netlist for RTL integration was generated. The VIVADO design suite 2018.2 was used for simulation and synthesis. The model was successfully dumped onto the Zynq7 – XC7Z010-1CLG400 device FPGA board. The PCG sensor data captured in FPGA internal Block RAM memory were passed through the pre-processing module to remove the unwanted noise. The cleaned data were then processed through the cardiac cycle separation engine to differentiate the systole and diastole cycles. The feature extraction module extracted the features mentioned in Table 1 from systole and diastole. The extracted six featured sets were passed through the ANN model to classify them into five heart sound component groups. The decision logic outputted the true-negative (TN), true-positive (TP), false-negative (FN), and false-positive (FP) for corresponding heart sound components. Figure 7 shows the FPGA hardware board developed for the proposed system.

The ANN with the inverse delayed neuron model was implemented in MATLAB^R^ using the neural network toolbox and the fixed-point toolbox as discussed in previous sections. The model was simulated to check for functionality and then used to calculate the synaptic weights and bias required for the hidden and output layers. The simulated fixed-point MATLAB model was taken as a golden reference for further hardware realization using FPGA.

**Figure 8:**
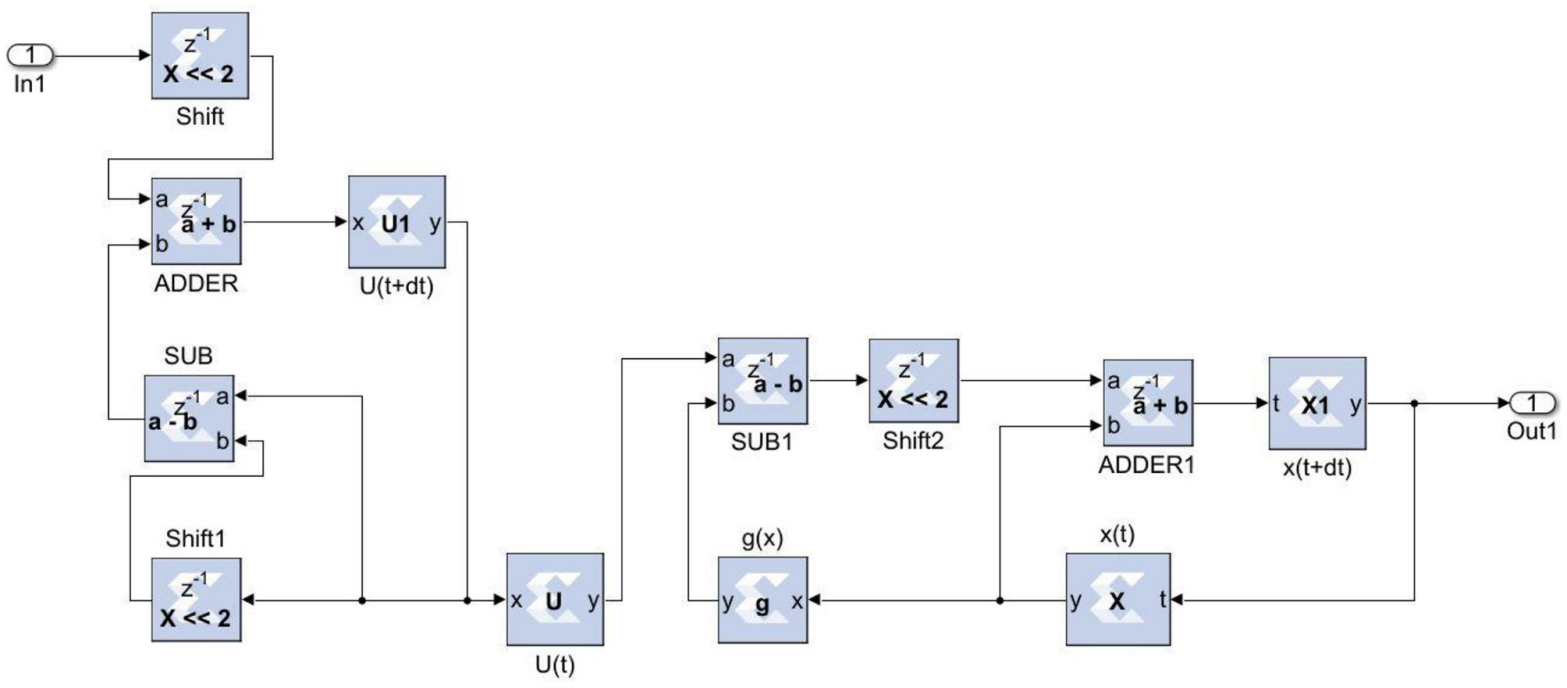
Neuron model.

The Xilinx System generator tool was used for the implementation and generation of the Verilog code for the system integration. As discussed in earlier sections, the ANN model was realized in three stages. For the first stage, in the input layer, the inputs were scaled with weights and passed to hidden neurons. For the second stage, the bias was added to the summed weights by the hidden layer, which then passed through the delayed activation function. For the third stage, the output layer computed the output value from all hidden neurons, the output bias, and the activation function. The sigmoid activation function was a building block that was used in both the hidden and output layers (26). Equation (16) was used to implement the inverse activation function, which consists of an adder, multiplier, and constant value. As shown in Figure 8, the neuron model was realized using a Mult-Add block and a constant block for bias, an activation function.

**Figure 9:**
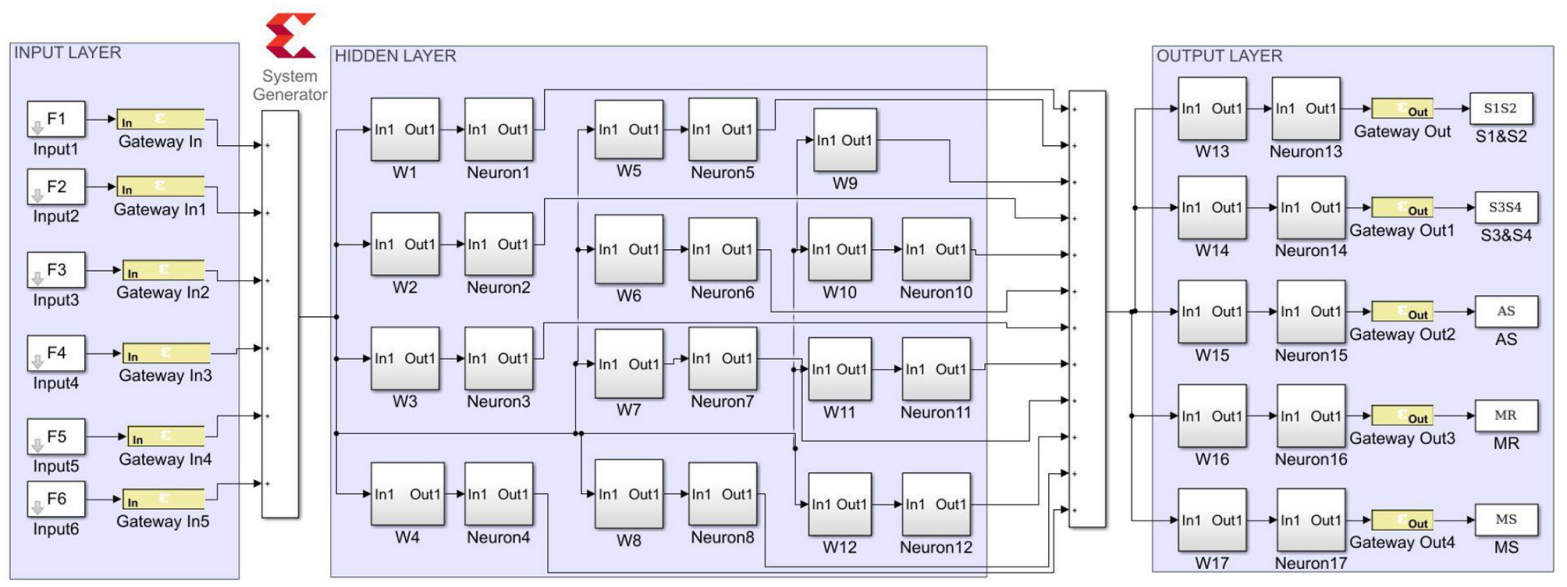
System generator implementation of the ANN model.

Figure 9 depicts the proposed ANN model implementation. The estimated sigmoid value is closer to the real sigmoid value obtained from MATLAB, allowing the approximation effect to be reduced for improved accuracy when implemented in hardware.

## Experimental Results and Discussion

The extraction of cardiac sounds from the MEMS-based high-performance phonocardiography system using a neural network was based on the ID function model of the neuron. The neural networks based on the ID function model and the conventional neuron models were realized on FPGA, and the hardware requirements and performance of the two models were compared. The proposed system was validated using the data of 30 patients in accordance with the Declaration of Helsinki. After obtaining informed consent, a total of 60 patients were made available for evaluation, with the data of 30 patients being used for training the neural networks and the data of the remaining 30 patients used for testing the proposed system.

**Figure 10:**
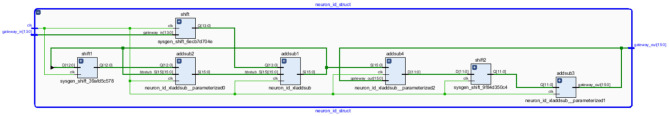
RTL Schematic—Neuron Model.

**Figure 11:**
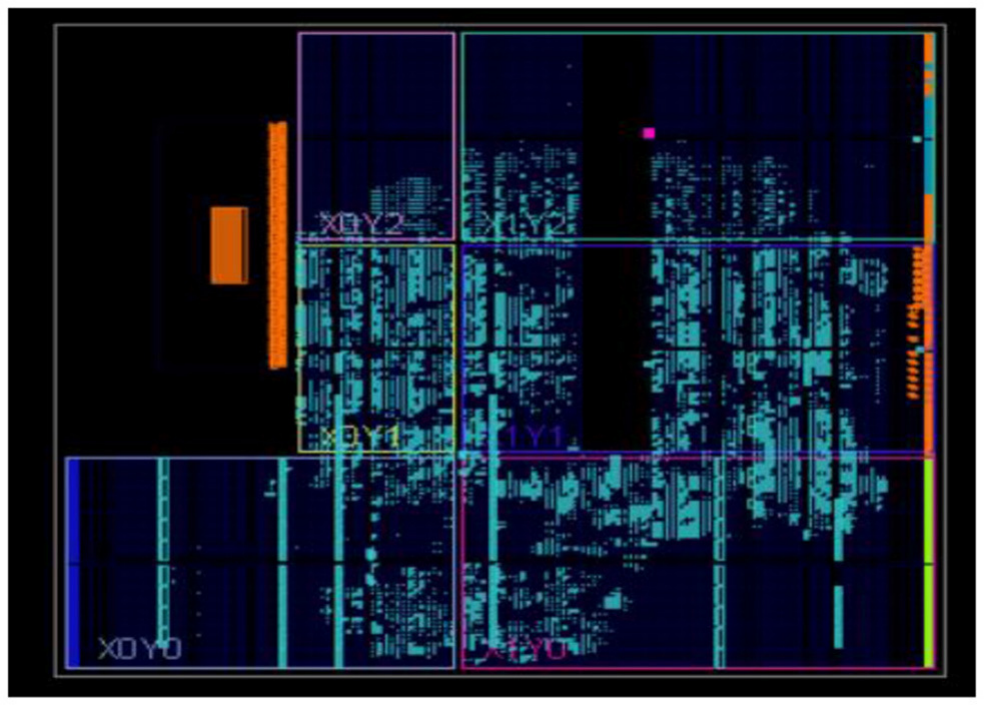
Physical layout for the proposed system.

The neural networks were trained offline using data from the 30 patients in MATLAB^R^, and then implemented on FPGA to reduce design circuitry. The MATLAB^R^ model weights and biases were used as hard-coded values in the FPGA ANN model to reduce computational cycles and achieve the accuracy obtained in the simulation. The FPGA implementation of the inverse tangent sigmoid function, which requires the realization of an N-shaped activation function, involved multiplication but not division. The multiplier was all that was required for the functional units of the ID model. This greatly reduced hardware complexity. The system generator FPGA netlist files were used to run the synthesis, and implement and generate the bit file needed to program the FPGA. Figure 10 depicts the RTL schematic for the neuron model following RTL synthesis. The physical layout of the proposed system is depicted in Figure 11.

**Figure 12:**
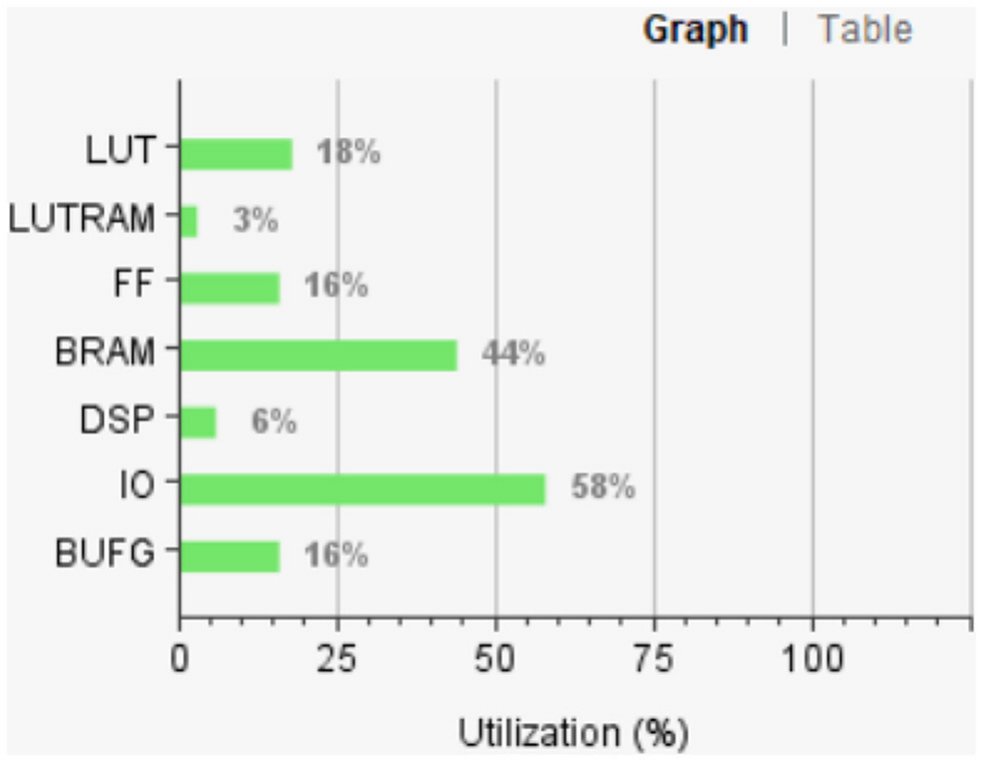
Resource utilization of the proposed system.

Figure 12 shows the FPGA resource utilization of the proposed layout after place and route.

**Figure 13:**
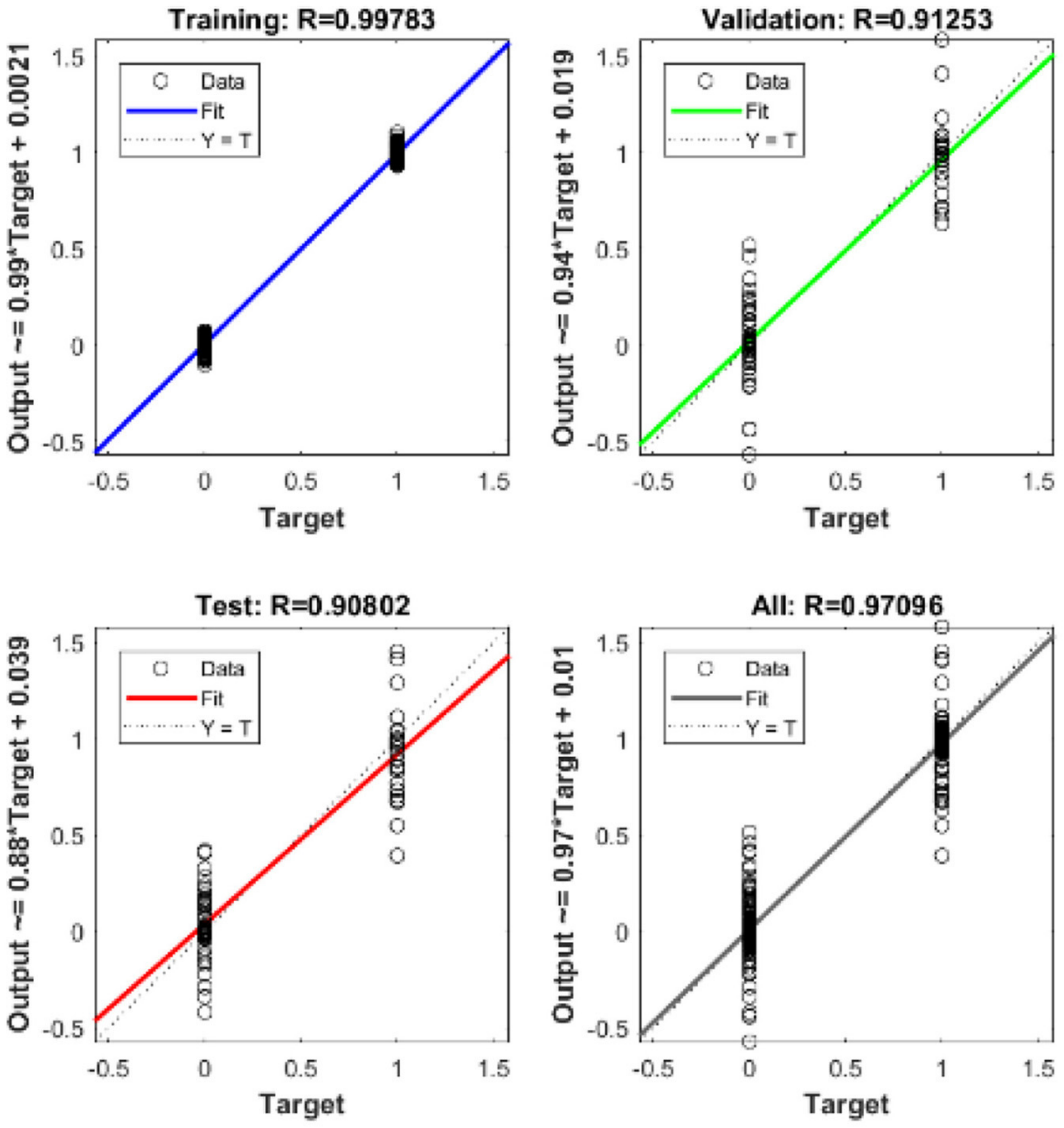
Regression analysis of neural network based on the ID function model of the neuron.

The mean square error in the extraction of cardiac sound components detection rate using neural networks with 12 neurons in the hidden layer was .9. Regression analysis was performed on the input and target datasets, and the mean square error was found to be 4.4 × 10^−5^. Figure 13 depicts the regression analysis of the training and validation of the network. In the regression analysis, the parameter “*R*” = 0.99 represents the correlation between extracted cardiac components and actual cardiac components.

**Figure 14:**
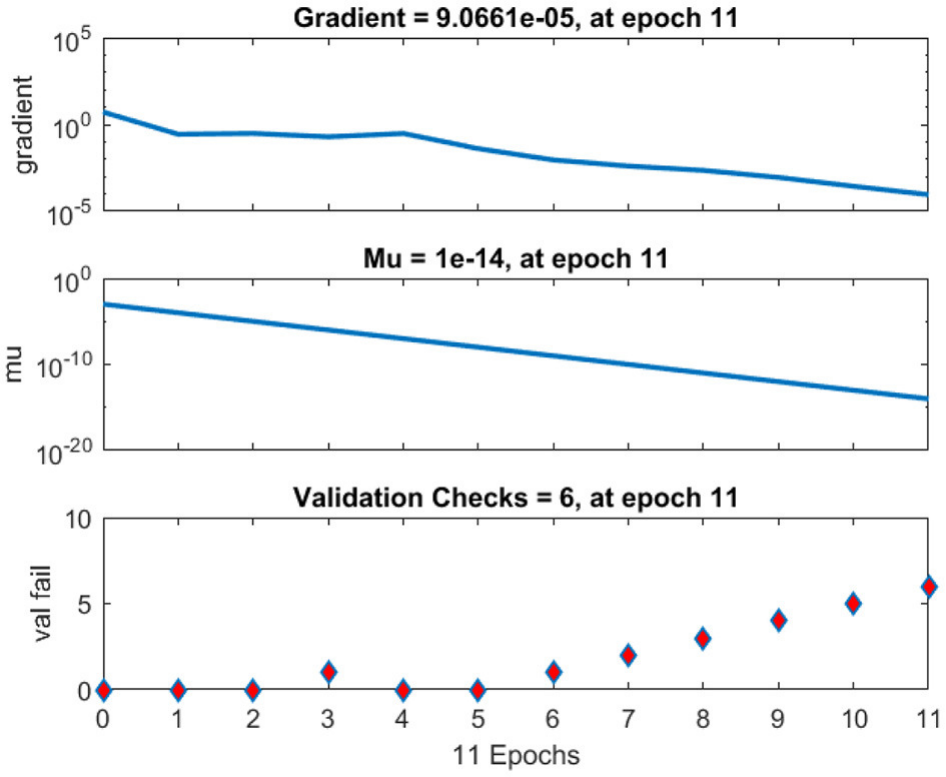
Training state analysis of neural network based on the ID function model of the neuron.

**Figure 15:**
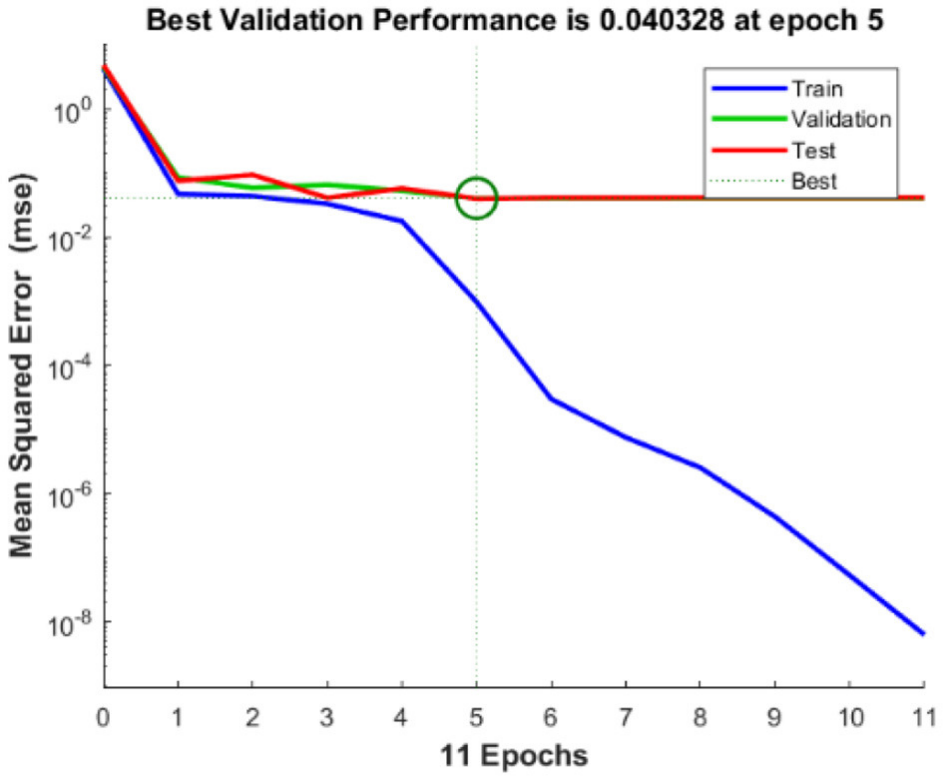
Performance analysis of neural network based on the ID function model of the neuron.

The regression analysis showing the training and validation of the network is depicted in Figure 13. The parameter “*R*,” which is equal to 0.99 in the regression analysis, signifies the correlation between extracted cardiac components with actual cardiac components. Figure 14 depicts the training state analysis of a neural network based on the ID function model of a neuron. Figure 15 depicts a neural network for performance analysis. Training analysis was performed for epoch 11, with a gradient factor of 9.0661 × 10^−5^ and validation checks equal to 6.

In accordance with the Declaration of Helsinki, clinical trials were conducted on 30 patients using the proposed high-performance phonocardiography system, and the results were compared to the known test results from the medical practitioner. The prevalence of disease in the tested population, the outcome of the diagnostic test, and the sensitivity and specificity of the test all had an impact on the reliability of any diagnostic test result. The sensitivity, the specificity rate, and the accuracy can be computed as follows


(17) $$\begin{array}{l} \text{Sensitivity }=\frac{\text{ TP}}{\text{TP}+\text{FN}}\times 100\%\; \end{array}$$

(18) $$\begin{array}{l} \text{Specificity }=\frac{\text{ TN}}{\text{TN}+\text{FP}}\times 100\%\; \end{array}$$

(19) $$\begin{array}{l} \text{Accuracy }=\frac{\text{ TP}+\text{TN}}{\text{TP}+\text{TN}+\text{FP}+\text{FN}}\times 100\text{\%} \end{array}$$

where true-negative (TN) represents the number of correct heart sound components rejected, true-positive (TP) represents the number of correct heart sound components detected by the proposed system, false-negative (FN) represents the number of incorrect heart sound components rejected, and false-positive (FP) represents the number of incorrect heart sound components detected by the proposed system. Table 2 displays the sensitivity, specificity, and accuracy of cardiac component detection for a healthy individual under specific disease conditions.

**Table 2:** Accuracy of proposed system for different cardiac components in heart sounds.

**Heart sound components**	**Sensitivity (%)**	**Specificity (%)**	**Accuracy**
S1 and S2	99.1	99.3	0.99
S3 and S4	98.1	98.6	0.98
Aortic stenosis	98.3	98.7	0.98
Mitral stenosis	98.5	98.7	0.98
Mitral regurgitation	98.2	98.4	0.98

**Figure 16:**
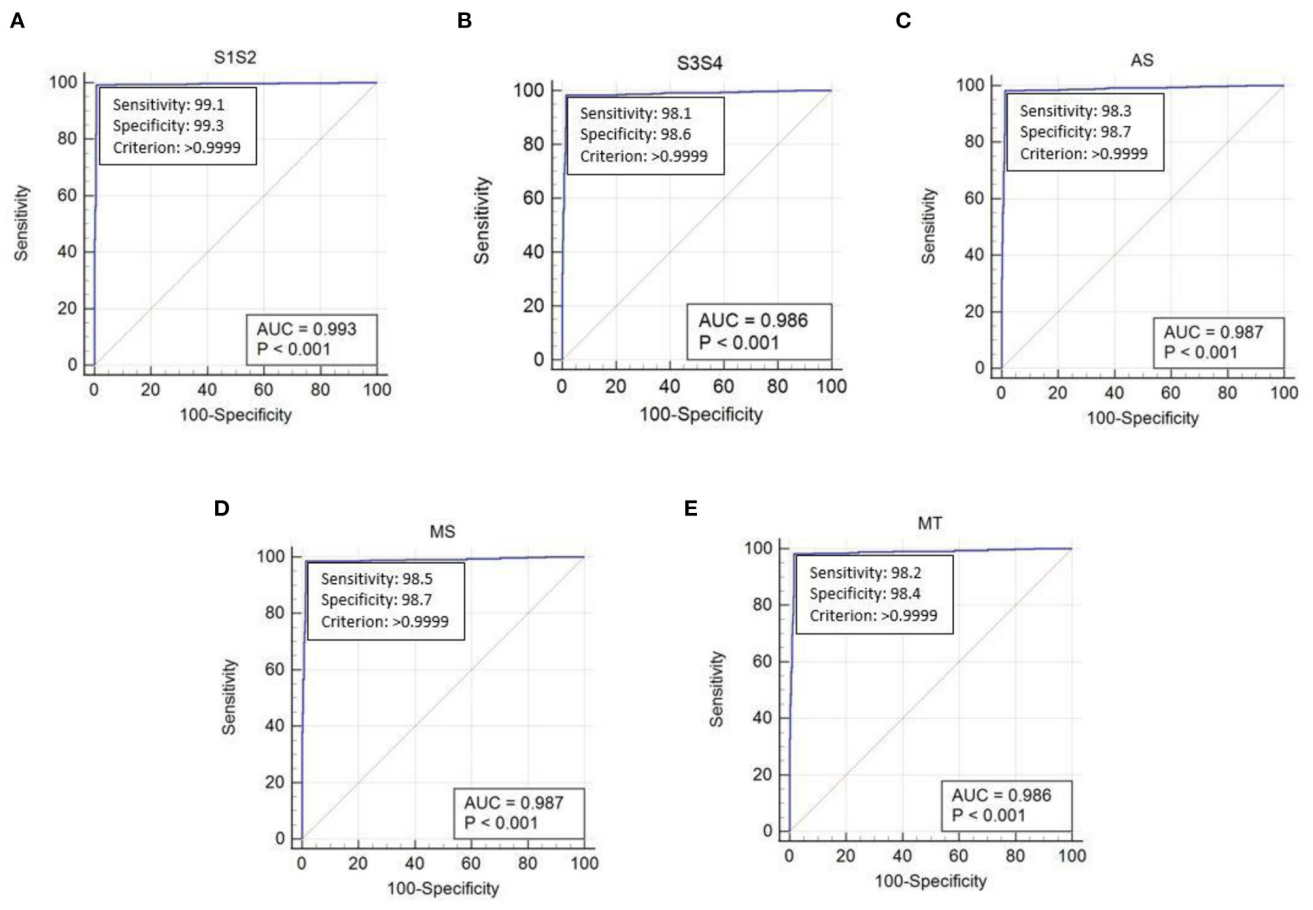
The receiver operating characteristic (ROC) curve analysis of heart sound components with ID function of the neuron model **(A)** first and second heart sound, **(B)** third and fourth heart sound, **(C)** aortic stenosis, **(D)** mitral stenosis, and **(E)** mitral regurgitation.

The performance of the proposed method was evaluated using the receiver operating characteristic (ROC) curve area under curve (AUC) value. This validated the extraction of cardiac components from the captured data using the proposed algorithm. The ROC curve for the extraction of cardiac component accuracy for the proposed ID neuron model system is shown in Figure 16. The accuracy of S1 and S2, S3 and S4, aortic stenosis, mitral Stenosis, and mitral regurgitation is 99.3, 98.6, 98.7, 98.7, and 98.6%, respectively, based on the AUC values in Figures 16A–E.

## Conclusion

The current research focused on the development of multi-channel MEMS-based phonocardiography system to capture heart sounds and process the acquired sample to remove unwanted noise and derive a feature set using wavelet transforms. Thereafter, the low frequency cardiac sounds were extracted using the ANN based on the ID function of the neuron model. The neural network was trained using real, known data, and the proposed system was tested using patient test data. The developed ANN-based phonocardiography system was useful to the physician for recognizing abnormal, low-frequency heart sounds with a simple diagnosis setup similar to the stethoscope and visualizing graphical data for better medical diagnoses. The performance of the phonocardiography system was evaluated using 2,150 cardiac cycles of PCG from a cohort of 30 patients with different pathophysiological conditions, resulting in a sensitivity of 99% and an accuracy of 0.9.

## Data Availability Statement

The datasets presented in this article are not readily available because of Non-disclosure Agreement with the patients. Requests to access the datasets should be directed to Madhubabu, mail.madhubabu@gmail.com.

## Ethics Statement

The studies involving human participants were reviewed and approved by Citizens Hospitals Hyderabad. The patients/participants provided their written informed consent to participate in this study.

## Author Contributions

All authors contributed in developing the proposed system, methodology, analyze the results, and manuscript editing.

## Conflict of Interest

The authors declare that the research was conducted in the absence of any commercial or financial relationships that could be construed as a potential conflict of interest.

## Publisher's Note

All claims expressed in this article are solely those of the authors and do not necessarily represent those of their affiliated organizations, or those of the publisher, the editors and the reviewers. Any product that may be evaluated in this article, or claim that may be made by its manufacturer, is not guaranteed or endorsed by the publisher.
